# Myosin isoform expressed in metastatic prostate cancer stimulates cell invasion

**DOI:** 10.1038/s41598-017-09158-5

**Published:** 2017-08-16

**Authors:** Ivan V. Maly, Tera M. Domaradzki, Victoria A. Gosy, Wilma A. Hofmann

**Affiliations:** Department of Physiology and Biophysics, University at Buffalo-State University of New York, Buffalo, NY 14214 USA

## Abstract

During metastasis, tumor cells migrate out of their original tissue to invade other organs. Secretion of exosomes and metalloproteases is essential for extracellular matrix remodeling, enabling migration through tissue barriers. Metastatic prostate cancer is differentiated by expression of the rare isoform A of the molecular motor myosin IC, however the function of this isoform remained unknown. Here we show that it contributes causatively to the invasive motility of prostate cancer cells. We found that the isoform associates with metalloprotease-containing exosomes and stimulates their secretion. While the data show that myosin IC is involved in prostate cancer cell migration, migration outside extracellular matrix *in vitro* proves little affected specifically by isoform A. Nevertheless, this isoform stimulates invasion through extracellular matrix, pointing to a critical role in secretion. Both the secretion and invasion depend on the integrity of the motor and lipid-binding domains of the protein. Our results demonstrate how myosin IC isoform A is likely to function in metastasis, driving secretion of exosomes that enable invasion of prostate cancer cells across extracellular matrix barriers. The new data identify a molecule suitable for a mechanistically grounded development into a marker and target for prognosis, detection, and treatment of invasive prostate cancer.

## Introduction

Migration of tumor cells out of their original tissue (metastasis) to invade other organs is the leading cause of mortality in cancer^1, 2^. With regard specifically to prostate cancer, early diagnosis and accurate determination of the metastatic and invasive qualities of a patient’s cancer is of great importance for enabling appropriate treatment and would lead to more favorable clinical outcomes^3–6^. Recently we have identified a promising molecule whose expression differentiated metastatic forms of prostate cancer, the rare isoform A of the molecular motor myosin IC. Myosin IC^7, 8^ (human gene product MYO1C in the HuGO nomenclature^9^) is thought to be a ubiquitous type of unconventional single-headed non-muscle myosin, and our earlier data^10–12^ established a ubiquitous expression of its isoform B. The recently discovered isoform A, in comparison, proved tissue-specific and not expressed in the normal prostate. *In-situ* tumors of the prostate similarly lacked this isoform, but distant and lymph-node metastatic samples expressed it. The function of this isoform in the prostate and prostate-derived cells, however, remained unknown.

Myosin IC is involved in the motility of the neuronal growth cones^13^, and recent work established a role for myosin IC in the motility of retinal and mammary epithelial cells *in vitro* ^14, 15^. The methods employed in these studies did not distinguish between the isoforms, and the cell types were not among the few identified as expressing isoform A^11^. Nonetheless, we reasoned that myosin IC and specifically isoform A may be involved in the migration of prostate cancer cells out of the primary tumor. The hypothesis is consistent with the nature of myosin IC as a molecular motor and the generally documented involvement of other non-muscle myosin types in cell locomotion^16^. Metastasis, however, contrasts with the unimpeded motility *in vitro* that has been studied vis-à-vis myosin IC in the growth cones and retinal and mammary cells. Essential for the process of metastatic invasion is secretion of exosomes (extracellular vesicles^17, 18^) and matrix metalloproteases^19^, which enables remodeling and enzymatic degradation of the extracellular matrix, making possible migration of the invading cells through tissue barriers^20, 21^. Earlier reports implicated myosin IC in intracellular trafficking and exocytosis of sodium channels and slit diaphragm proteins in kidney cells^22, 23^, as well as glucose transporters in adipocytes^24, 25^. Although the secretion in these documented instances was not part of the exosomal secretion pathway, we reasoned by analogy and hypothesized that myosin IC and specifically isoform A may be involved in secretion of exosomes in prostate cancer cells, enabling their migration through the extracellular matrix.

Here we show that isoform A of myosin IC associates with exosomes and stimulates their secretion. Furthermore, the data demonstrate that myosin IC is involved in prostate cancer cell migration. Although migration outside extracellular matrix *in vitro* proves little affected specifically by isoform A, this isoform stimulates invasion through extracellular matrix, pointing to a critical role of its secretory function for the complex process of migration under the physiological conditions.

## Results and Discussion

### Myosin IC and isoform A in exosomes

**Figure 1:**
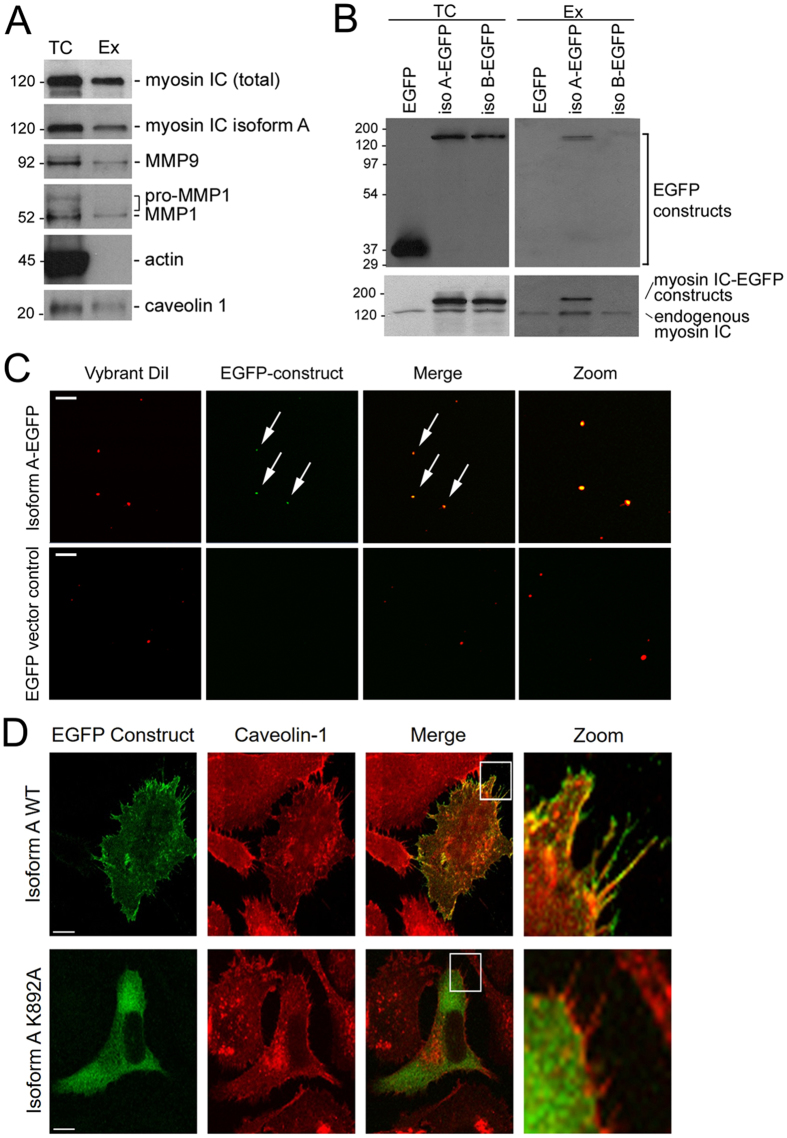
Localization of myosin IC isoform A in prostate cancer cells and exosomes. (**A**) Western blot analysis of total cell (*TC*) extract and secreted exosomes (*Ex*) of PC3 cells. Left labels: molecular weight in kDa. Right labels: antibody targets. (**B**) Western blot analysis of total extract and exosomes of PC3 cells transfected with EGFP-tagged isoforms A and B of myosin IC or with EGFP alone. Top panels: probed with anti-EGFP antibody. Bottom panels: probed with anti-myosin IC (non-isoform specific) antibody. (**C**) Fluorescence microscopy images of purified exosomes of PC3 cells transfected with EGFP-tagged isoform A or EGFP alone. Membrane visualized with Vybrant DiI dye. *Arrows*: vesicles displaying colocalization of the two fluorescent signals. *Zoom*: central area of image to the left, magnified. (**D**) Relative localization of EGFP-tagged isoform A with caveolin 1 in PC3 cells. Caveolin is visualized by indirect immunofluorescence. *WT*, wild type. K892A, substitution in the lipid-binding domain. *Scale bars* in (**C**,**D**) – 10 µm.

To begin characterizing the function of myosin IC in metastatic prostate cancer cells, we set out to test whether it is involved in secretion of exosomes. In support of the hypothesis, myosin IC was found in exosomes secreted by the cultured metastatic prostate cancer cell line PC3 (Fig. 1A). This finding is consistent with the proteomic identification of myosin IC in prostate-derived exosomes in urine^26^. In the exosomes we also detect MMP1 and MMP9, the interstitial and basement-membrane collagenases associated with prostate cancer cell invasivity and metastasis^20, 27–32^. The presence of their shorter mature form^19, 33, 34^ alongside the longer proenzyme (Fig. 1A) is consistent with a matrix-degrading functionality of these exosomes. Caveolin 1, the membrane component of exosomes that is also overexpressed in prostate cancer^21, 35, 36^, confirms the identification of the vesicles. This membrane-associated cytosolic protein has been previously identified as a marker of exosomes secreted by PC3 cells^35^. At the same time, the absence of actin is indicative of the purity of the exosome fraction from other cellular components. Significantly, the exosomes contain isoform A of myosin IC (Fig. 1A) – the rare isoform that is specifically expressed in metastatic prostate cancer^12^.

Selectivity of the exosome secretion pathway for isoform A is demonstrated in cells transfected with EGFP fusions of two isoforms of myosin IC (Fig. 1B): while both isoform A and isoform B are present in the total cell extract of PC3 cells, only traces of isoform B are detected alongside the abundant isoform A in the secreted exosomes. Exogenously expressed EGFP alone (not fused with the myosin) is similarly excluded from the exosomes, confirming the selectivity of secretion and the purity of the collected exosomes from any cell debris. Dual-wavelength fluorescence imaging of the exosomes stained with a membrane dye reveals that the vesicles containing isoform A represent a significant subset (Fig. 1C).

Microscopic localization of isoform A is consistent with a role in motility-supporting secretion. The EGFP-labeled construct of this isoform accumulates on the cell periphery in immediate proximity to the cell boundary and inside plasma membrane protrusions (Fig. 1D), where it colocalizes with caveolin 1. A substitution in the lipid-binding domain (K892A) that is known to abolish binding of myosin IC to phospholipids^37^ abrogated the peripheral localization of isoform A, causing an accumulation of the mutant form in the cell’s interior (Fig. 1D).

### Myosin IC in exosome secretion

**Figure 2:**
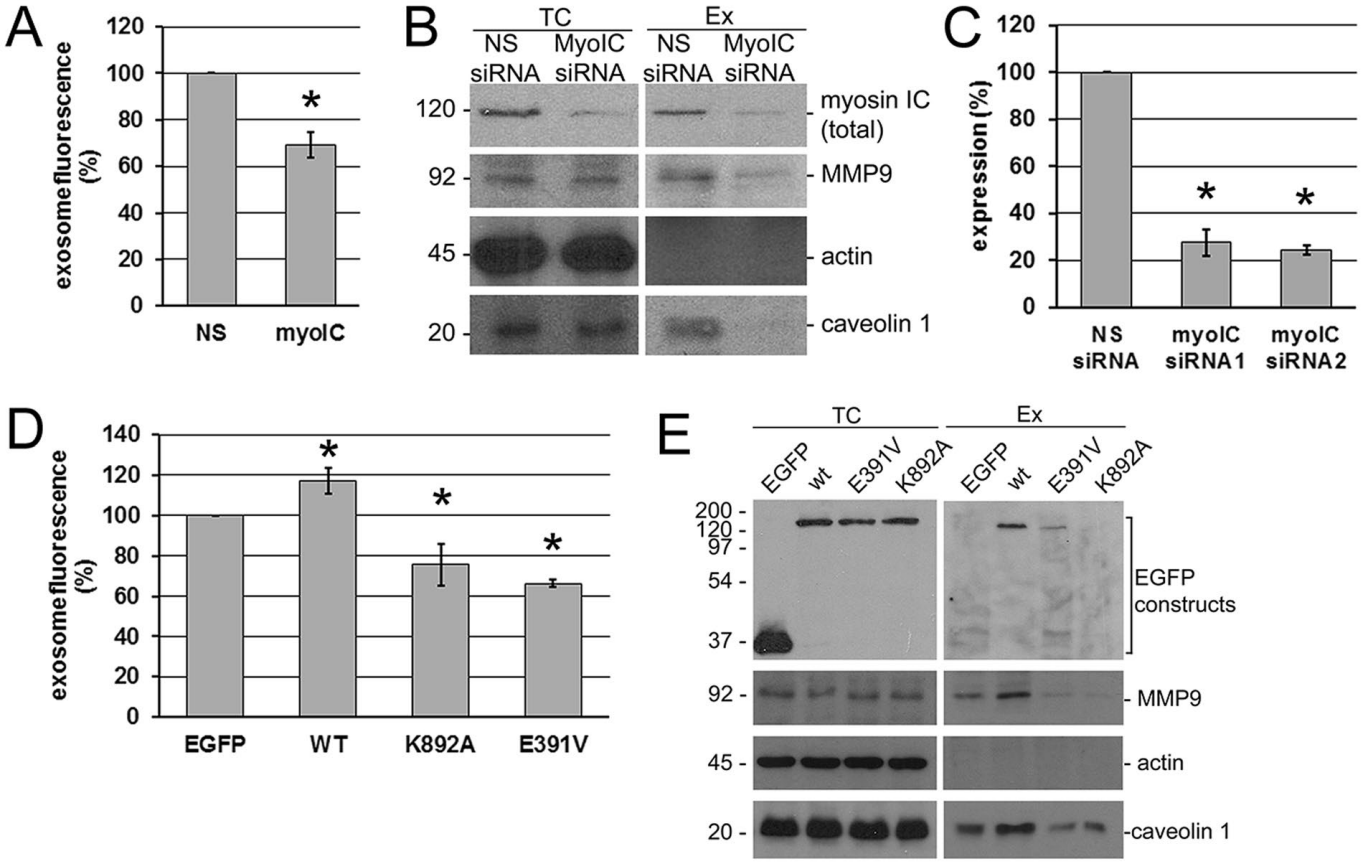
Effects of myosin IC and isoform A on exosome and metalloprotease secretion by prostate cancer cells. (**A**) Fluorescence of the secreted exosomal fraction (stained with membrane dye Vybrant DiI) from PC3 cells transfected with nonspecific (*NS*) and myosin IC-targeted (*myoIC*) siRNA. Data were normalized to the control (nonspecific siRNA treatment) in each experiment. N = 8 cell cultures per group. Experiment was repeated 4 times. Error bar: 95% c.i. on the mean. (**B**). Western blot analysis of total cell (*TC*) extract and secreted exosomes (*Ex*) from the siRNA knockdown experiments. (**C**) Quantification of the knockdown. N = 2 cell cultures per group. Experiment was repeated 2 times. Error bar: 95% c.i. on the mean. Two different sequences were targeted by siRNA 1 and 2; siRNA 1 was used in all other experiments shown. *Significant (p < 0.0001) deviations from the control. (**D**) Fluorescence of the secreted exosomal fraction measured as in *A*, from PC3 cells transfected with EGFP fusion constructs of myosin IC isoform A. *EGFP*, control transfection with EGFP alone. *WT*, wild-type isoform A. *K892A*, lipid-domain substitution. *E391V*, motor-domain substitution. Data presentation as in (**A**). N = 5 cell cultures per group. Experiment repeated 5 times. Each p-value in pairwise comparisons with the control is <0.0001 (*), and all null hypotheses are rejected at familywise significance level 0.05 if regarded as a multiple comparison using the Holm-Bonferroni correction. (**E**) Western blot analysis of total cell (*TC*) extract and secreted exosomes (*Ex*) from the EGFP fusion experiments.

To characterize the functional contribution of myosin IC to exosome secretion in prostate cancer cells, we measured the fluorescence of the isolated exosomes stained with a membrane dye. In PC3 cells transfected with myosin IC-targeting siRNA, the exosome secretion was suppressed compared with cells transfected with scrambled nonsense siRNA (Fig. 2A). The data show a knockdown of the myosin IC expression by the targeted siRNA (Fig. 2B,C), demonstrating a direct correlation between its expression and the secretion function in the cells. The measurement of the membrane fluorescence in the isolated exosomes is confirmed by the concomitant reduction of the caveolin 1 and MMP9 (Fig. 2B) content of the exosomes, pointing at the functional significance of the secretion reduction for the cells’ capacity for matrix degradation. The observed effect may be the result of the partial knockdown of the total myosin IC; alternatively, it could stem more specifically from the knockdown of one or more of the isoforms that are simultaneously targeted by the siRNA.

To overcome the limitation of the siRNA knockdown whereby the sequence distinguishing the isoforms is too short to be targeted and to probe further the hypothesized involvement of isoform A in the secretion function, we analyzed secretion in cells transfected with various isoform A constructs. Using the same fluorescence method, the exosome secretion was found to be elevated in cells expressing the EGFP fusion of wild-type isoform A, compared with the treatment control consisting of cells transfected with EGFP alone (Fig. 2D). This shows functionality of specifically isoform A in driving the exosome secretion in prostate cancer cells, and the preservation of this functionality in the experimental fusion construct with EGFP. At the same time, substitutions in either the lipid-binding (K892A) or the motor domain (E391V) resulted in a decrease of the exosome secretion (Fig. 2D). Taken together with the localization data for the lipid-binding mutant and co-fractionation with caveolin 1, the mutant analysis demonstrates dependence of the secretion on the lipid-mediated association with the exosomal vesicles. As the substitution in the motor domain that is utilized in these experiments has been previously shown to inhibit myosin IC-mediated intracellular motion^38^, the results also indicate that isoform A’s activity as a molecular motor contributes mechanically to the exosome secretion.

Analysis of the secreted exosomes (Fig. 2E) shows a greatly reduced presence of the motor-domain mutant form, while the lipid-binding mutant is undetectable in this fraction. As expected under the hypothesis, the caveolin and MMP9 content of the exosomes changes in line with the fluorescence proxy for the exosome count, displaying an elevation in the wild-type experiment and a reduction in both mutant experiments.

### Myosin IC in prostate cancer cell motility

**Figure 3:**
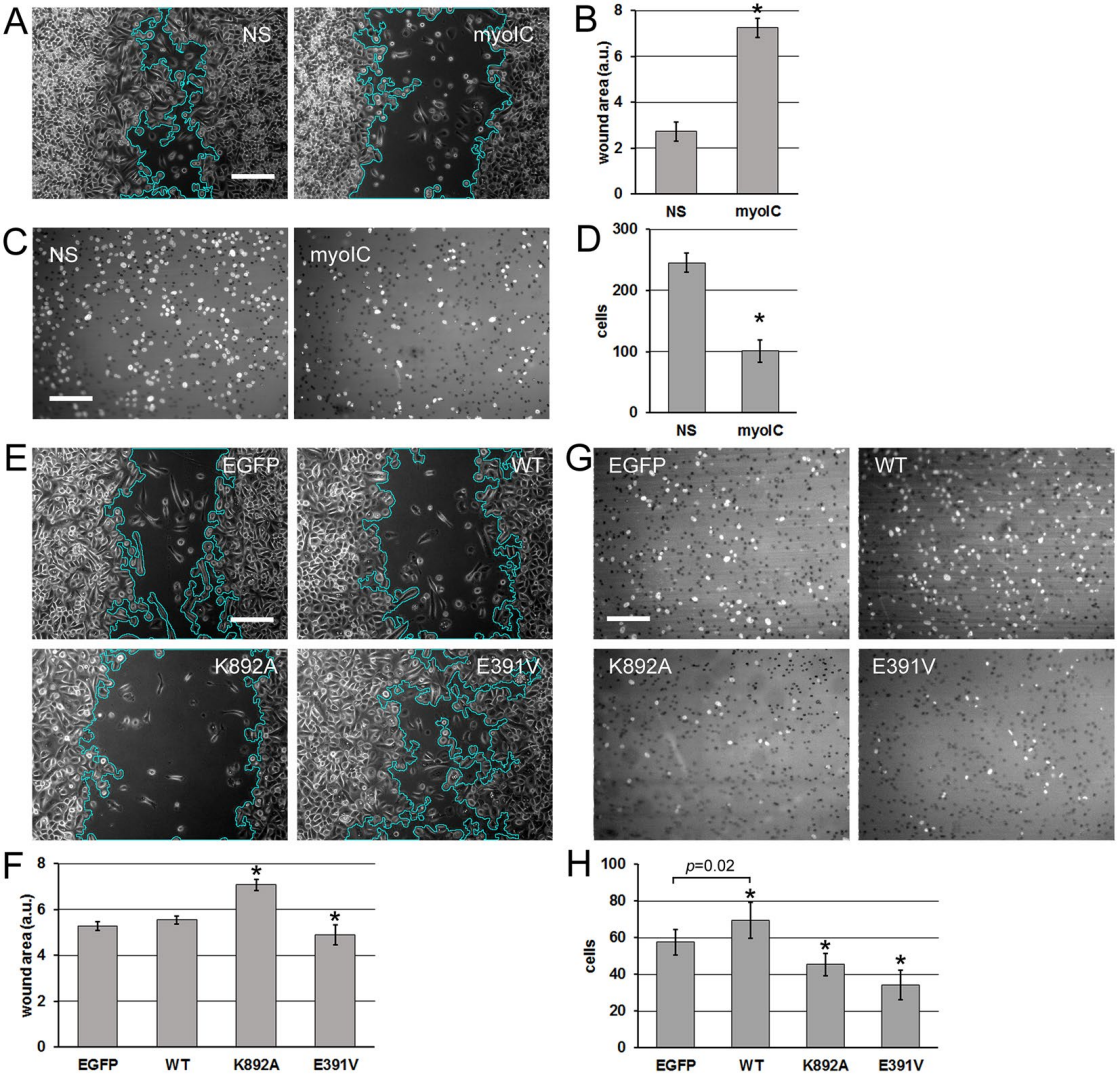
Effects of myosin IC and isoform A on motility and invasion of prostate cancer cells. (**A**) *In-vitro* wound closure experiment on PC3 with the siRNA knockdown. Scratched and healing cell monolayers were photographed in phase contrast 24 h after scratching. Representative fields are shown. Wound area outline in cyan is overlaid by the software used for the automated measurement. (**B**) Quantification of the experiment in *A*. Error bars: 95% c.i. on the mean. N = 40 camera fields per group. Experiment reproduced 4 times. (**C**) Matrigel invasion experiment on PC3 cells with the siRNA knockdown. Nuclei of cells that have invaded across the reconstituted extracellular matrix layer were visualized by DAPI staining and fluorescence microscopy. Representative fields are shown. (**D**) Quantification of the experiment in (**C**). Error bars: 95% c.i. on the mean. N = 36 camera fields per group. Experiment reproduced 4 times. (**E**). *In-vitro* wound closure experiment on PC3 cells expressing the isoform A constructs or EGFP as the treatment control. Visualization as in (**A**). (**F**) Quantification of the experiment in (**E**). Error bars: 95% individual c.i. on the mean. N = 52 camera fields per group. Experiment repeated 4 times. (**G**) Matrigel invasion experiment on PC3 cells expressing the isoform A constructs. Visualization as in (**C**). (**H**) Quantification of the experiment in (**G**). Error bars: 95% individual c.i. on the mean. *Significant (p < 0.05) deviations from the control. One-tailed p-values in pairwise comparisons with the EGFP control are: WT – indicated on the graph, K892A – 0.005, E391V – <0.0001. If regarded as a multiple comparison, each null hypothesis of equality to the control is rejected on the familywise significance level 0.05 using the Holm-Bonferroni correction. *Scale bars* in (**A**,**E**,**C**,**G**) – 200 µm.

The role of myosin IC in the motility of prostate cancer cells was examined using the knockdown of its expression with siRNA. The *in-vitro* “wound” assay^39, 40^, which involves scratching a monolayer of cells grown to confluence, showed that the cells with the knockdown migrate into the experimental wound more slowly than cells subjected to mock treatment with scrambled nonsense siRNA (Fig. 3A,B). The result is consistent with the involvement of myosin IC in the free locomotion of retinal and mammary epithelial cells^14, 15^. In the normal mammary cells, the reduction of free locomotion was connected with a reduction of cell adhesion and spreading, and linked to a loss of contact inhibition^15^. It is likely that the migration of the transformed prostate epithelial cells involves the same mechanisms.

Having established the applicability to the prostate cancer cells of the general function of myosin IC in cell locomotion, we probed specifically the invasive motility involved in metastasis. To this end, we counted the cells that migrate through a layer of reconstituted basement membrane (matrigel – primarily collagen IV) in the transwell assay^41, 42^. More cells transfected with nonsense siRNA invade across the matrigel layer compared to cells transfected with the anti-myosin IC siRNA (Fig. 3C,D). The result is consistent with the effect of myosin IC knockdown on secretion of metalloproteases (Fig. 2B). The direct effect of the knockdown on free migration, however, supplied an alternative explanation of the invasion experiment that tests the combined effect of migration and matrix digestion.

To shed more light on the question, we repeated the migration and invasion assays using cells transfected with the wild-type and mutant forms of isoform A. Compared with EGFP transfection as a treatment control, overexpression of wild-type isoform A does not affect the free migration substantially (Fig. 3E,F). Juxtaposed with the sensitivity of the process to reduction of the total myosin IC expression (Fig. 3A,B), this result indicates that either the migration function is saturated at the endogenous level of expression or it does not reside with isoform A. The dominant-negative effect of the lipid-binding mutant (K892A, Fig. 3E,F) supports the former conclusion. In this connection, it bears repeating that the endogenous expression of isoform A is not normal but characterizes metastatic prostate cancer cells^12^.

**Figure 4:**
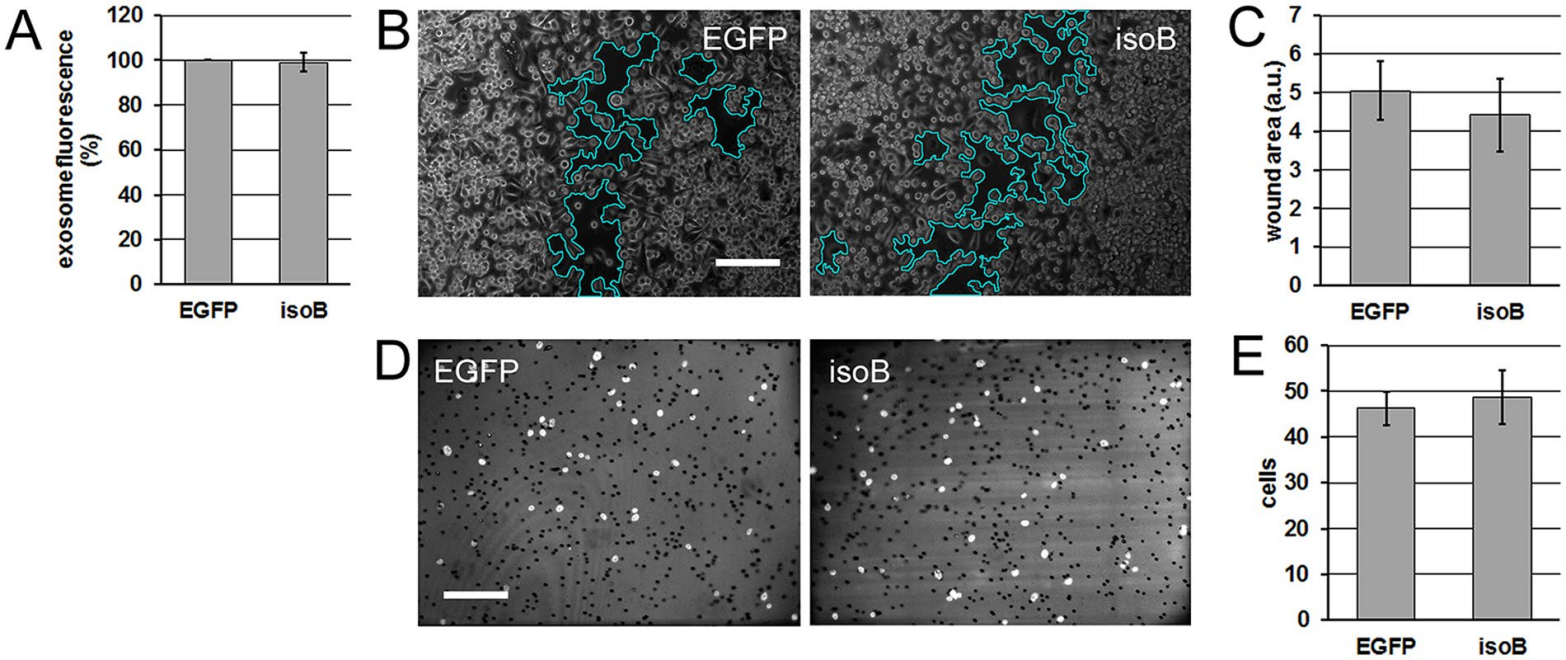
Exosome secretion, motility and invasion of cells overexpressing isoform B. (**A**) Fluorescence of the secreted exosomal fraction (stained with membrane dye Vybrant DiI) from PC3 cells transfected with EGFP alone (control transfection, *EGFP*) and EGFP fusion of myosin IC isoform B (*isoB*). Data were normalized to the control. N = 3 cell cultures per group. Experiment was repeated 3 times. Error bar: SE. (**B**) *In-vitro* wound closure experiment on PC3 transfected with the same constructs. Scratched and healing cell monolayers were photographed in phase contrast 24 h after scratching. Representative fields are shown. Wound area outline in cyan is overlaid by the software used for the automated measurement. (**C**) Quantification of the experiment in (**B**). Error bars: SE. N = 10 camera fields per group. Experiment reproduced 2 times. (**D**) Matrigel invasion experiment. Nuclei of cells that have invaded across the reconstituted extracellular matrix layer were visualized by DAPI staining. Representative fields are shown. (**E**) Quantification of the experiment in (**D**). Error bars: SE. N = 18 camera fields per group. Experiment reproduced 2 times. *Scale bars* in (**B**,**D**) – 200 µm.

The lack of a dominant-negative effect of the motor-domain mutant in the wound assay (E391V, Fig. 3E,F) is inconclusive as to the role of isoform A in unimpeded locomotion, but proves informative in juxtaposition with the matrigel assay data (Fig. 3G,H). Here we observe enhanced invasive ability of the PC3 cells overexpressing the wild type and an inhibited invasion of the cells expressing the mutant forms. Like the siRNA invasion data (Fig. 3C,D), these effects are directly correlated with the secretion data (Fig. 2), which is consistent with the hypothesis that isoform A-driven secretion of exosomes determines the invasive potential of prostate cancer cells. While the inhibition of invasion by the lipid-binding domain mutant (K892A) may be attributed to this form’s dominant-negative effect on free migration (Fig. 3E,F), the comparison of the two assays (Fig. 3E,F vs. GH) shows that the invasion enhancement by overexpression of the wild type is unrelated to cell locomotion per se. The same can be observed about the inhibition of invasion through matrigel by the motor-domain substitution (E391V). Thus, both the increase and the decrease in the invasive capacity can be attributed to the changes in the isoform A-driven exosome secretion. Further supporting this attribution, overexpression of isoform B did not significantly alter exosome secretion, cell migration, or cell invasion (Fig. 4).

## Concluding remarks

The results establish the mechanism whereby the recently discovered isoform A^10^ of the molecular motor myosin IC contributes to the prostate epithelial cells’ secretion of exosomes, enhancing their ability to invade across extracellular matrix barriers, such as the basement membrane whose breaching marks the transformation of the benign prostatic neoplasia into a metastasizing carcinoma^43–46^. In connection with the cells’ basement membrane breaching capacity *in vivo*, our findings concerning the MMP9 content of the exosomes is of special relevance. Along with the interstitial collagenase MMP1 that we find alongside it, this basement-membrane collagenase has been associated with prostate cancer cell invasivity and metastasis^20, 27–32^. The new evidence for a role in secretion is also consistent with cell motility due to lipid raft recycling^14^.

The presence of myosin inside the exosome can be explained in the framework of the mechanism and topology of the exosome formation by invagination into larger intracellular vesicles^47^. A protein attached to the cytosolic side of such a membrane will be found after invagination on the inner surface of the vesicle inside the forming multivesicular endosome. Subsequent to the emptying of the multivesicular endosome and the release of its contents from the cell, this location will correspond to the interior of the secreted exosome. Similarly, a protein attached to the cytoplasmic side of the plasma membrane will be found inside the exosome that buds off directly from the plasma membrane^47^. While this would also be true for a protein attached passively, the documented role of myosin IC in exocytosis^22–25^ suggests an active role, whereby the myosin may generate the force needed for the membrane motility and dynamics involved in the intracellular transport and invagination, via its interaction with the filaments of the actin cortex. In this connection, the positive correlation of the expression of the filamentous actin regulator cortactin with exosome secretion^48^ may suggest a functional relationship to myosin IC. The actin-based invadopodia, which have been shown to be the sites of exosome secretion in squamous carcinoma cells^49^, can be examined as likely sites of the hypothetical myosin IC action on the cortex.

Beyond the immediate effect on the invasive ability of the prostate epithelial cells that is documented here, the secretion function of this molecule is likely to have a secondary promoting effect on invasion via the exosome-mediated stimulation of the matrix remodeling by the cells of the tumor stroma^50^, and on the other known mechanisms of exosome-mediated communication^21, 51–53^ in the tumor microenvironment. Taken together with this isoform’s specific expression in invasive forms of prostate cancer^12^, the new data shed additional light on prostate carcinogenesis and identify isoform A of myosin IC as a molecule suitable for a mechanistically grounded development into a marker and target for prognosis, detection, and treatment of invasive prostate cancer.

## Methods

### Cell culture

PC-3 cells were purchased from ATCC (American Type Culture Collection, Manassas, VA, cat. No. CRL-1435) and cultured in RPMI 1640 medium (Corning Life Sciences, Corning, NY) supplemented with 10% fetal bovine serum and 1% penicillin/streptomycin, at 37 °C with 5% CO_2_. The cell line has not been authenticated by our laboratory or tested for mycoplasma.

### Antibodies

The myosin IC-isoform A antibody is a mouse monoclonal antibody that was raised against the myosin IC isoform A specific N-terminal peptide and recognizes exclusively myosin IC isoform A^10^. The total myosin IC antibody recognizes a region in the tail domain that is common to all myosin IC isoforms (Santa Cruz Biotechnology, Dallas, TX). Other antibodies used: α-β-actin (Sigma-Aldrich, St Louis, MO); α-GFP (EMD Millipore; Billerica, MA); α-caveolin-1 (Cell Signaling Technologies, Danvers, MA); α-MMP1 (EMD Millipore; Billerica, MA), α-MMP9 (EMD Millipore; Billerica, MA); peroxidase-conjugated as well as Texas Red-conjugated secondary anti-mouse or anti-rabbit antibodies (Jackson ImmunoResearch Laboratories, West Grove, PA).

### Exosome isolation and analysis

**Figure 5:**
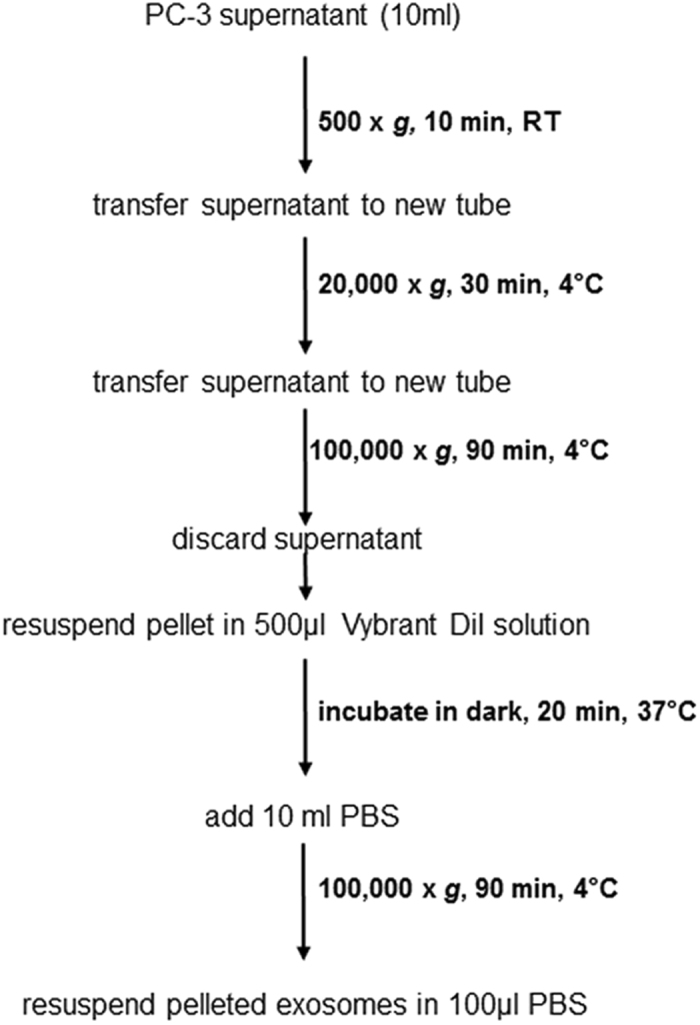
Exosome isolation protocol.

For exosome isolation 0.8 × 10^6^ cells were plated in 10cm tissue culture dishes in complete culture medium. 16 h after plating, medium was exchanged for serum free RPMI 1640. After 24 h, the supernatant was collected, cleared of cell debris and subjected to serial ultracentrifugation for exosome isolation^35^ with some modifications. Figure 5 shows a schematic of the centrifugation protocol used for exosome isolation. Isolated exosomes were stained with Vybrant DiI (Thermo Fisher Scientific Inc., Waltham, MA) solution that was prepared in PBS (phosphate buffered saline) according to manufacturer’s instructions.

Vybrant DiI fluorescence was measured using a BioTek Synergy Microplate Fluorescence microplate reader (BioTek Intruments, Inc., Winooski, VA). Fluorescence measurements of isolated exosomes were adjusted to cell number.

For microscopic analysis, isolated Vybrant DiI stained exosomes were mixed on a coverslip with Prolong Gold antifade reagent (Cell Signaling Technology, Inc. Danvers, MA) and mounted. Images were taken on a confocal microscope (LSM 510 Meta; Carl Zeiss, Inc.) using 63 × NA 1.40 Plan-Apochromat objectives and processed using Photoshop (Adobe).

### Plasmids, siRNAs, and transfections

The wild-type myosin IC-EGFP constructs used were derived previously^10^. The amino acids were numbered as before^54^, from the beginning of the sequence common to the myosin IC isoforms, i.e. excluding the leading 35 amino acids that are unique to isoform A. Point mutations were generated using the Quick Change II site-directed mutagenesis kit (Stratagene, La Jolla, CA).

Validated siRNA that targets specifically myosin IC (total) (Silencer Select #s9199) as well as the non-specific (NS) siRNA that was designed for, and used at the same concentration as its respective gene-targeting siRNA were purchased from Thermo Fisher (Carlsbad, CA). In the knockdown quantification experiment, both the above siRNA was used and Silence Select #s9201.

For transfection, cells were plated either on cover glasses in tissue culture plates for fluorescent analysis or directly in tissue culture dishes. 16h after plating, cells were transfected using Lipofectamine 2000 (Thermo Fisher, Carlsbad, CA).

### Cell Growth and Viability

Following transfections, cell growth and viability were quantified by cell counting after Trypan Blue (Hyclone, Marlborough, MA) addition using an automated cell counter (TC20, Bio-Rad, Hercules, CA). Cell viability was calculated as: (number of live cells divided by number of live plus dead cells) × 100.

### Total cell extract preparation and immunoblotting

Total cell extract was prepared as described^12^. For detection, proteins in total cell extract from an equal number of cells or from vesicles were separated by SDS-PAGE and transferred onto nitrocellulose membrane. After the transfer, the blots were either probed intact or cut according to size of the respective probed protein and probed with specific antibodies. The immunoreactive bands were detected by enhanced chemiluminescence. Densitometry for expression quantification was performed in Image J (National Institutes of Health, Bethesda, MD). Myosin bands were normalized to the actin in the same lanes.

### Immunofluorescence staining and confocal microscopy

Immunofluorescence staining on PC-3 cells was performed essentially as described^10^. Coverslips were mounted with Prolong antifade containing 4′,6′-diamino-2-phenylindole (DAPI) (Cell Signaling Technologies, Danvers, MA). Images were taken on a confocal microscope (LSM 510 Meta; Carl Zeiss, Inc.) using 63 × NA 1.40 Plan-Apochromat objectives and processed using Photoshop (Adobe).

### Migration assay

The *in-vitro* wound healing assay^39, 40^ was employed to assess the capacity of cells for locomotion unimpeded by extracellular matrix. The cells were seeded in 6-well plates (manufacturer) at equal densities, and after 24 h transfected as described above. 24 h after transfection with myosin constructs, or 48 h after transfection with siRNA, the cell monolayers were scratched with a micropipette tip, producing a “wound” which was clear of cells and had a uniform width narrower than the microscope’s camera’s field of view. Before imaging, the culture was washed with PBS, and fresh medium was added. Nonoverlapping fields covering the length of the wound were acquired on an inverted microscope (DMIL, Leica, Wetzlar) using a CCD camera (RT Slider, SPOT Imaging, Sterling Heights, MI). The wound area (area free of cells) in each field was detected automatically and measured in the Image J software using the MRI Wound Healing Tool utility. Identical algorithm parameters were used on all images.

### Invasion assay

The Matrigel modified Boyden chamber (“transwell”) assay^41, 42^ (Corning, Bedford, MA) was employed to assess the capacity of cells for invasion across extracellular matrix barriers. The assay was set up according to the manufacturer’s instructions. Cells were seeded in wells at equal densities 24 h after transfection with myosin constructs or 48 h after transfection with siRNA. Following a 24 h incubation, the matrigel and cells remaining on the top surface of the well bottom were removed by scraping, and the cells on the bottom surface were fixed in 3% paraformaldehyde in PBS. The porous membrane forming the well bottom was separated from the well and mounted in the DAPI-containing medium as described above for fluorescent visualization of the nuclei. Nonoverlapping fields covering the area of the membrane were acquired on the same camera as above on a Leica DMRE microscope. The nuclei in each field were detected automatically and counted in Image J using the Analyze Particles utility, and the number was accepted as the cell count. Identical algorithm parameters were used on all images.

### Statistical analysis

Exosome secretion measurements were normalized to the control in each experiment, then aggregated by treatment across the replicate experiments. Cell motility measurements were aggregated by treatment. In each case, the sample mean and the 95% confidence interval on the mean were calculated for graphical presentation, and p-values determined with the null hypothesis of equality to the control and the directional alternative. For the normalized data, the equality of the mean to 100% was tested directly using the confidence interval; for the non-normalized data, the equality to the control mean was tested using the method of sampling distribution of difference between means. Where the control served as a comparison for multiple treatments (in experiments with myosin constructs), the pairwise p-values were further assessed against the Holm-Bonferroni correction for multiple comparisons, setting the critical familywise false discovery rate at 0.05.
